# Bowtie Nanoantenna LSPR Biosensor for Early Prediction of Preeclampsia

**DOI:** 10.3390/bios14070317

**Published:** 2024-06-24

**Authors:** Ke Yi, Mengyin Ao, Ting Ding, Danxi Zheng, Lin Li

**Affiliations:** 1 Gynecology Department of West China Second University Hospital, Sichuan University, Chengdu 610041, China; 2 Key Laboratory of Birth Defects and Related Diseases of Women and Children, Ministry of Education, Sichuan University, Chengdu 610041, China

**Keywords:** localized surface plasmon resonance, bowtie nanoantenna, preeclampsia, early prediction

## Abstract

Objective: The concentration of the placental circulating factor in early pregnancy is often extremely low, and the traditional prediction method cannot meet the clinical demand for early detection preeclampsia in high-risk gravida. It is of prime importance to seek an ultra-sensitive early prediction method. Methods: In this study, finite-different time-domain (FDTD) and Discrete Dipole Approximation (DDA) simulation, and electron beam lithography (EBL) methods were used to develop a bowtie nanoantenna (BNA) with the best field enhancement and maximum coupling efficiency. Bio-modification of the placental circulating factor (sFlt-1, PLGF) to the noble nanoparticles based on the amino coupling method were explored. A BNA LSPR biosensor which can specifically identify the placental circulating factor in preeclampsia was constructed. Results: The BNA LSPR biosensor can detect serum placental circulating factors without toxic labeling. Serum sFlt-1 extinction signal (Δλmax) in the preeclampsia group was higher than that in the normal pregnancy group (14.37 ± 2.56 nm vs. 4.21 ± 1.36 nm), *p* = 0.008, while the serum PLGF extinction signal in the preeclampsia group was lower than that in the normal pregnancy group (5.36 ± 3.15 nm vs. 11.47 ± 4.92 nm), *p* = 0.013. The LSPR biosensor detection results were linearly consistent with the ELISA kit. Conclusions: LSPR biosensor based on BNA can identify the serum placental circulating factor of preeclampsia with high sensitivity, without toxic labeling and with simple operation, and it is expected to be an early detection method for preeclampsia.

## 1. Background

Currently, there has been a notable delay in the age at which women are marrying and having children, resulting in an increased risk of advanced maternal age, preeclampsia, neonatal asphyxia, premature delivery, and low birth weight infants [1]. Preeclampsia (PE) is a unique and common serious complication during pregnancy, and is the main cause of morbidity and death of mother and child [2]. World Health Organization (WHO) findings show that the maternal mortality rate due to preeclampsia and eclampsia is about 16% in developed countries and as high as 25% in developing countries [1,3]. More than half of preeclampsia-related maternal deaths can be prevented by prophylactic use of low-dose aspirin before 16 weeks of pregnancy, but this has no significant effect after 16 weeks of pregnancy [4,5,6]. It can be seen that early prediction of preeclampsia is crucial to reduce maternal mortality. Traditional prediction methods, including maternal risk factors (detection rate of 37.0% under 5% false positive rate) [7], mean arterial pressure (detection rate of 49.3% under 10% false positive rate) [8], and ultrasonic uterine and placental blood flow parameters combined with maternal risk factors (detection rate of 59% under 5% false positive rate) [9] cannot meet the demand for early clinical recognition of preeclampsia well. Therefore, it is extremely important to seek an ultra-sensitive early prediction method.

Patients with preeclampsia may exhibit abnormalities in placental circulatory factor secretion prior to the onset of clinical symptoms, enabling the early prediction of preeclampsia risk through monitoring maternal placental circulatory factor alterations, thereby offering insights for timely intervention [10,11,12,13]. Serum placental circulating factors, specifically soluble vascular endothelial growth factor receptor 1 (sFlt-1) and placental growth factor (PLGF), have been identified as potentially significant markers for predicting early-onset preeclampsia in the first and second trimesters of pregnancy. The sFlt-1/PLGF ratio has demonstrated clinical utility in the short-term prognostication of preeclampsia, with a notably high negative predictive value of 99.3% for cases where the sFlt-1/PLGF ratio is ≤38, indicating a low likelihood of developing preeclampsia within one week [14,15]. The low concentration of serum placental circulating factor in early pregnancy necessitates the use of highly sensitive detection methods.

In recent years, the rapid advancement of surface plasma optics has emerged as a promising technical approach for the early detection of diseases. Surface plasma is collective oscillation of free electrons near metal–dielectric interfaces, which are collected by incident electromagnetic waves. There are two forms of surface plasmons. One is propagating surface plasmon or surface plasmon polaritons (SPPs) and the other is localized surface plasmon (LSPR). The SPPs propagate at interfaces as waveform at the interface between dielectric and a metal film. The LSPR is confined surface plasmons at interfaces of metallic nanoparticles or nanostructures, which creates strong local electromagnetic fields at interfaces of metallic nanostructures. This process involves the conversion of biological signals into optical signals through the resonance effect between incident light and the free electron gas mass present on the surface of metal nanostructures [16,17]. The LSPR is very sensitive to the refractive index. A small change of refractive index of the surface will result in a significant change of wavelength of resonance in an LSPR spectrum. The utilization of the refractive index sensitivity and field-enhancement characteristics of localized surface plasmon resonance (LSPR) sensing has garnered significant attention in recent years due to its ability to achieve super sensitivity. When exposed to light irradiation of specific wavelengths, the coupling effect of LSPR leads to the generation of a locally enhanced electromagnetic field on or near the surface of metal nanostructures. This phenomenon enables the surpassing of the traditional diffraction limit, as light in free space becomes confined to the subwavelength range [18,19,20]. For the bowtie nanoantenna (BNA) structure, its sharp tips and specific geometric parameters help achieve resonance at specific wavelengths, and optimizing the far-field scattering is aimed at achieving better sensing performance. The manipulation of the material, geometric parameters, and dielectric environment of the BNA structure allows for effective regulation of its LSPR characteristics [21,22]. Additionally, a significant increase in the electric field is observed in the nanogap region, highlighting the BNA structure’s potential for application in optical biosensing [23,24,25]. This capability makes it particularly well-suited for the sensitive detection of disease markers.

This study aims to closely integrate nanotechnology, biosensing, analytical chemistry and clinical medicine. The BNA structure with the best field enhancement and maximum coupling efficiency is developed, and the modification methods of bowtie nanoparticle surface placental circulating factor (sFlt-1, PLGF) are explored. The BNA LSPR biosensing principal verification experiment system was established to specifically identify placental circulating factors of preeclampsia, and the feasibility of the BNA optical sensor based on LSPR for early prediction of preeclampsia was explored.

## 2. Methods

### 2.1. Reagents and Consumables

Anti-human sFlt-1 monoclonal antibody, human recombinant sFlt-1 protein, anti-human PLGF monoclonal antibody and human recombinant PLGF protein were all purchased from SAB (Greenbelt, MD, USA). 11-mercaptoundecanoic acid (11-MUA), n-hydroxysuccinimide (NHS) and 1-(3-dimethylaminopropyl) -3-ethylcarbodiimide (EDC) were purchased from Sigma-Aldrich (St. Louis, MO, USA). Ethanolamine, glycine, HCL and anhydrous ethanol were all purchased from Kelon (Chengdu, China). The sFlt-1 and PLGF ELISA kits were purchased from R&D Systems (Minneapolis, MN, USA). PBS buffer and PBST buffer (0.01 M, pH = 7.4) were purchased from AMRESCO (Solon, OH, USA). The ultra-pure water (18.2 MΩ/cm^2^) was prepared by Millepore ultra-pure water Machine (Boston, MA, USA). All reagents are analytically pure.

### 2.2. Patients and Sample Collection

The procedures adhered to ethical standards established by the Human Trial Committee of the hospital, were approved by the committee, and involved obtaining informed consent from subjects for participation in the clinical study. Eligible participants in this study were pregnant women at high risk who chose to receive prenatal care and deliver at the teaching hospital. Inclusion criteria for participation included a gestational age of less than 16 weeks at the initial prenatal visit, a singleton pregnancy and the presence of at least one specified risk factor such as positive anti-phospholipid antibody, a BMI of 35 kg/m^2^ or higher, or comorbid conditions such as diabetes, chronic hypertension, kidney disease, autoimmune disease, diastolic blood pressure of 80 mmHg or higher during the first prenatal examination, a family history of preeclampsia, primiparity and age younger than 18 years or older than 40 years. Two milliliters of peripheral blood were obtained from pregnant women at 24–28 weeks gestation and anticoagulated with EDTA in a collection vessel. The supernatant was promptly centrifuged within 4 h of collection and stored at −80 °C for future analysis. Serum samples from the pregnant women were analyzed using bowtie LSPR biosensor and ELISA. Pregnancy outcomes were monitored, with participants categorized into either the preeclampsia group or the control group based on the development of preeclampsia.

### 2.3. Simulation Design and Preparation of Bowtie Nanostructures

The characteristics of metal nanoparticle arrays were analyzed using numerical methods such as finite difference time domain (FDTD) and discrete dipole approximation (DDA). FDTD is a method of modeling optical properties of nanophotonic devices by solving Maxwell’s equations. In the FDTD simulation, the unit cell was used to model the periodic array of the BNAs. The light incident normally conformed to the surface of the BNAs. The permittivity of gold used for the simulation was adopted from the study of Johnson and Christy [26]. The DDA is a method to compute scattering and absorptions of electromagnetic waves by particles. The DDA method is preferred to calculate optical properties of nanoparticles. By considering the periodic and small size of the BNAs, FDTD and DDA were combined to simulate the BNAs. The sensitivity of nanostructures was optimized by varying periods and sizes and the configuration to yield the most pronounced response characteristics was identified. By integrating DDA and FDTD algorithms, an analysis was conducted on the factors influencing the sensitivity of the sensing structure. The material composition, shape, and arrangement of the metal nanostructure were modified based on the local coupled electric field strength, with the aim of designing BNA structures that exhibit the most robust LSPR response. This study offers a theoretical foundation and empirical evidence to support the fabrication of nanostructures.

The BNAs were produced using electron beam lithography (EBL). The primary technical methods for producing dimers of metal nanoparticles include gold sputtering, polymethyl methacrylate (PMMA) spin coating, EBL exposure, development, electron beam evaporation of gold and cleaning.

### 2.4. The BNA LSPR Sensing Experimental Verification System

The optical test platform was constructed for the purpose of analyzing the spectrum of the nanoparticle array and investigating the characteristic peak of LSPR. The optical detection unit utilizes an Ocean Optics USB4000 (Shanghai, China) optical fiber spectrometer equipped with a CCD detector. The light source employed is the DH-2000 UV-Vision-near-infrared light source, which emits non-polarized light. All spectral measurements are conducted within the confines of the standard geometric transmission path. The data-processing unit utilizes the spectrum software (OceanView) from Ocean Optics (Dunedin, FL, USA) for image acquisition, and the Origin data-processing software version 9.0 (Northampton, MA, USA) for denoising and analyzing the acquired data.

### 2.5. Preparation of Biosensitive Membranes

**Figure 1:**
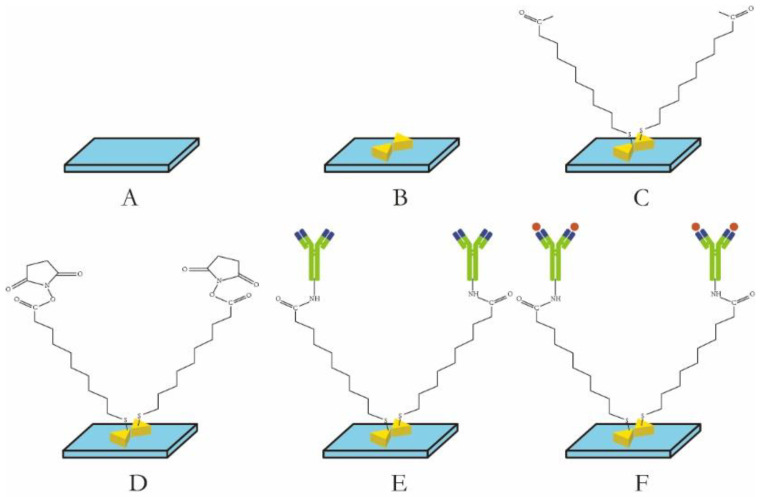
Schematic diagram of sensor chip preparation: (**A**) Glass base; (**B**) bowtie nanoparticles; (**C**) MUA monolayer; (**D**) EDC/NHS activated surface carboxyl groups; (**E**) monoclonal antibody against target protein; (**F**) detection of target proteins.

The ionic bond formation between the sulfhydryl end and the metal nanoparticles on the surface of the nanoparticles was achieved using an amino coupling method to create an MUA self-assembly layer (Figure 1). Additionally, an amide bond was formed between the carboxyl end and the amino end of the antibody. The experimental conditions, including the concentration of the coupling agent and the incubation time, were optimized to determine the most effective preparation scheme for the biosensitive layer. Following a 9 h immersion of the metal nanochips in 1 mM MUA at room temperature, the nanochips underwent a cleaning process involving anhydrous ethanol for 5 min, repeated twice, and ultra-pure water for 5 min, once, at a rotational speed of 100 RPM, followed by drying with nitrogen. Subsequently, the chips were immersed in a mixed solution containing 75 mM EDC.HCL, 15 mM NHS and other specified volumes at room temperature for 2 h to activate the surface carboxyl group. This was followed by a washing step with ultra-pure water for 2 min, repeated three times, and drying with nitrogen. A 50 μL amount of a 10 ug/mL solution of anti-human sFlt-1/PLGF monoclonal antibody was added to the chip and incubated at 4 °C for 10 h. The chip was then washed twice with PBS for 5 min each and once with ultra-pure water for 5 min. It was dried with nitrogen gas and stored in a refrigerator at 4 °C for future use.

### 2.6. Detection Target

#### 2.6.1. Recombinant sFlt-1/PLGF Standard Test

The concentration gradient of sFlt-1/PLGF standard protein solution (10 pg/mL, 100 pg/mL, 1 ng/mL, 10 ng/mL, 100 ng/mL, 1 µg/mL, 10 µg/mL) was analyzed by conducting at least three tests for each standard concentration. Mean and standard deviation of the test values for each concentration standard were obtained using OriginPro 9.0 software, and a standard curve was generated along with the corresponding curve equation. The LSPR spectrum undergoes changes following repeated testing (at least 10 times) of the reaction involving the sFlt-1/PLGF diluter. The standard deviation ∆λmax is determined as the detection noise value, with the minimum effective response signal being set at three times the noise value. Regarding peak processing, we maintain consistency in our approach, averaging three detections for target identification.

#### 2.6.2. Serum Samples Were Detected by LSPR Sensor Chip and ELISA

The LSPR metal nano-immune sensor chip was allowed to equilibrate to room temperature after being removed from the refrigerator at 4 °C for 30 s. The serum sample, stored at −80 °C, was thawed at room temperature and also equilibrated to room temperature. Subsequently, 50 uL of the sample was added to the sensor chip’s surface and placed in a constant temperature incubator at 37 °C for 30 min. The sensor chip was cleaned twice with PBST for 5 min each time, followed by a single cleaning with ultra-pure water for 5 min, and dried with nitrogen. Each sample was measured three times and the results were averaged. The ELISA kit was utilized for the detection of serum samples, with each specimen undergoing double-hole detection in strict adherence to operational instructions. The optical density (OD) was quantified using an enzyme-labeled instrument (BIORAD 680) at a wavelength of 450 nm. Figure 1E illustrates the utilization of an LSPR nanosensor chip immobilized with anti-sFlt-1/PLGF monoclonal antibody to detect 10 ng/mL recombinant human sFlt-1/PLGF protein, with the calculation of the spectral peak redshift value (Δλmax). The LSPR Δλmax was determined through the detection of serum samples using both LSPR sensor and ELISA methodologies.

## 3. Results

### 3.1. Design and Preparation of Bowtie Antenna Nanoparticles

**Figure 2:**
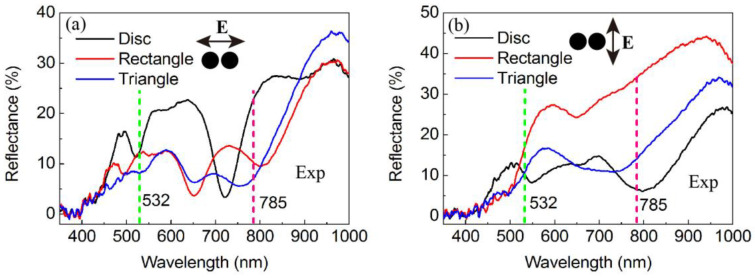
The reflectance spectra of the dimeric nanocrystals were measured with polarization in both the x direction (**a**) and y direction (**b**). The green and pink lines on the spectra correspond to excitation wavelengths of 532 nm and 785 nm, respectively.

The sensitivity of the sensing structure was analyzed through the utilization of DDA and FDTD algorithms, which involved modifying the material composition, shape, and structural arrangement of metal nanostructures based on the local coupled electric field strength. This approach aimed to design a BNA structure with optimized shape and feature size to achieve the strongest LSPR response. As illustrated in Figure 2, the empirical data demonstrates that the BNA configuration exhibits significant field enhancement, attributed to the amalgamation of the local surface plasmon resonance of the nanoantennas with their image modes within the gold film. This approach to field enhancement circumvents the conventional difficulty associated with fabricating exceedingly minute nanotips or nanogaps, offering a straightforward method for developing sophisticated substrates characterized by expansive surface areas, heightened sensitivity and enhanced dependability.

**Figure 3:**
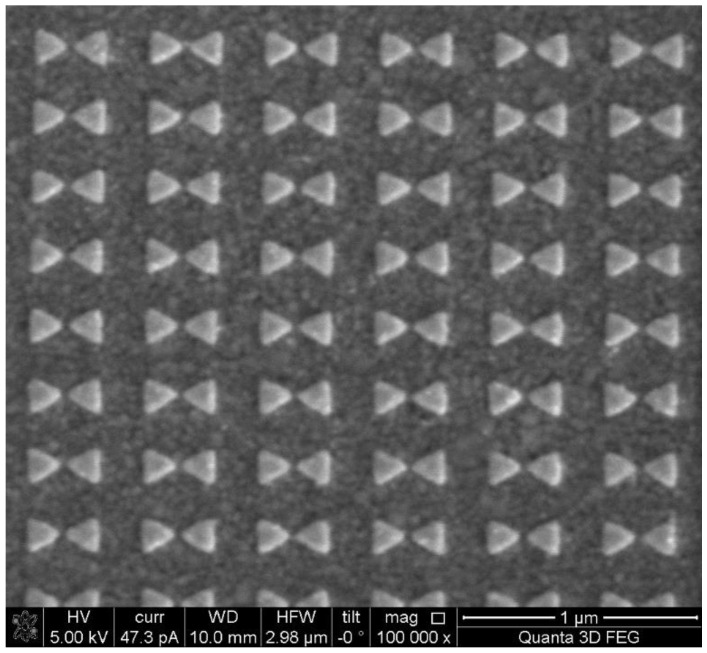
SEM image and structure diagram of bowtie nano.

The nano-optical antenna’s triangular bowtie structure, known for its simplicity in preparation and strong local optical field enhancement, guided the nano-fabrication process based on simulation outcomes. Electron beam lithography (EBL) was utilized to precisely manipulate the size, shape, pattern and spacing of particles on the glass substrate. The plasma bowtie nanodimer on the Au film featured a 20 nm gap, as illustrated in Figure 3. The bowtie nanoparticle array was fabricated using EBL, and its morphology was examined through electron microscopy.

### 3.2. Sensors Detect Placental Circulating Factors in Serum of Preeclampsia Patients

**Figure 4:**
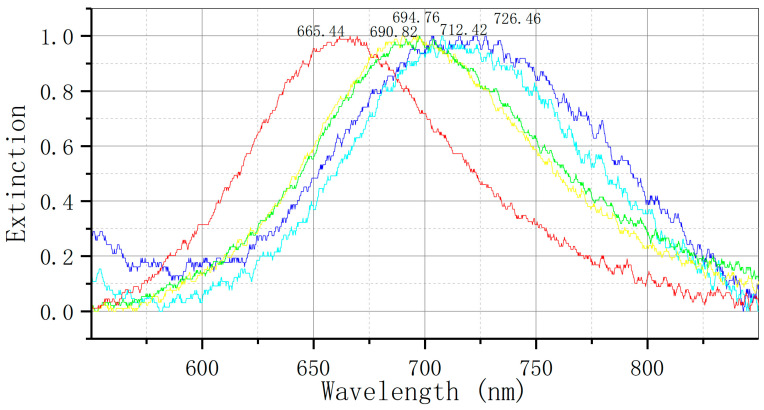
Detection of sFlt-1 recombinant protein by bowtie nanoscale LSPR biosensor: red: nude; yellow: MUA; green: EDC/NHS; light blue: anti-sFlt-1 monoclonal antibody; dark blue: sFlt-1 recombinant protein (Δλ = +14.04 nm).

The study utilized a constructed LSPR bowtie sensor chip to analyze the levels of serum placental angiogenesis-related cytokines (sFlt-1 and PLGF) in high-risk pregnant women between 24 and 28 weeks of gestation. Participants were categorized into a preeclampsia group and a normal single pregnancy group based on their pregnancy outcomes. The aim of the study was to assess the potential of the LSPR biosensor for early prediction of preeclampsia. As illustrated in Figure 4, the bowtie nanoscale LSPR biosensor, immobilized with serum anti-sFlt-1 monoclonal antibody, successfully detected recombinant sFlt-1 protein, resulting in a spectral peak redshift of +14.04 nm.

**Figure 5:**
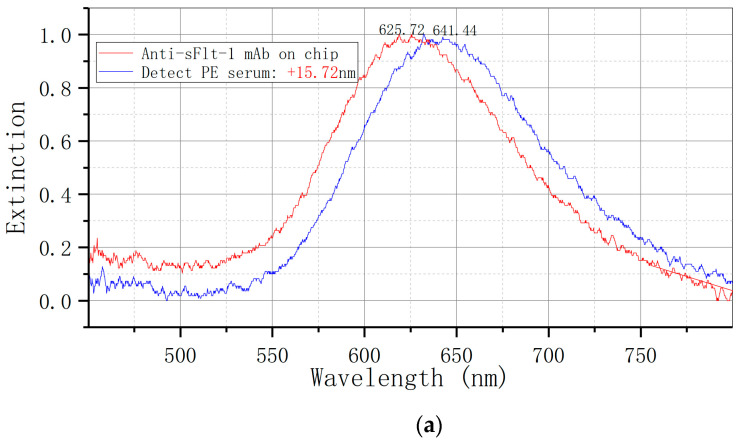

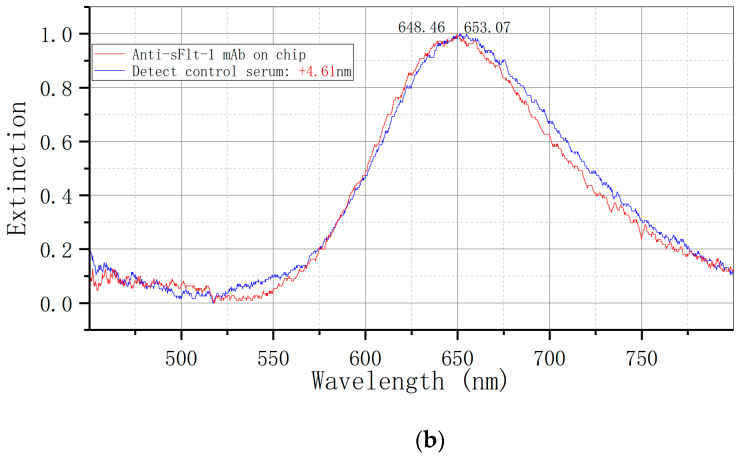
Detection of serum sFlt-1 expression by LSPR bowtie sensor: (**a**) PE group (Δλ = +15.72 nm); (**b**) control group (Δλ = +4.61 nm). mAb: monoclonal antibody.

The levels of serum sFlt-1 were measured in two cohorts of pregnant women using a bowtie LSPR biosensor. The results, illustrated in Figure 5, indicate a redshift in the elimination peak from 625.72 nm to 641.44 nm (Δλ = +15.72 nm) in the group with preeclampsia, and from 648.46 nm to 653.07 nm (Δλ = +4.61 nm) in the control group.

**Figure 6:**
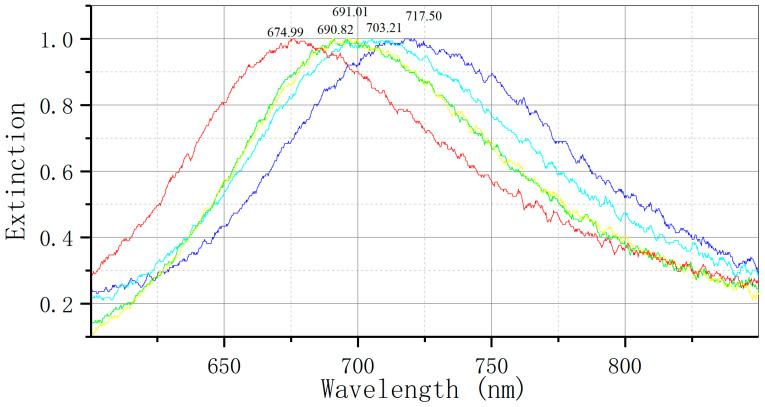
Bowtie nanoscale LSPR biosensor for detection of PLGF recombinant protein: red: nude; yellow: MUA; green: EDC/NHS; light blue: anti-PLGF monoclonal antibody; dark blue: PLGF recombinant protein (Δλ = +14.29 nm).

As illustrated in Figure 6, the bowtie nanoscale LSPR biosensor, equipped with a fixed serum anti-PLGF monoclonal antibody, demonstrates the ability to detect recombinant PLGF protein and elicit a spectral peak redshift of +14.29 nm, thereby facilitating accurate and efficient detection.

**Figure 7:**
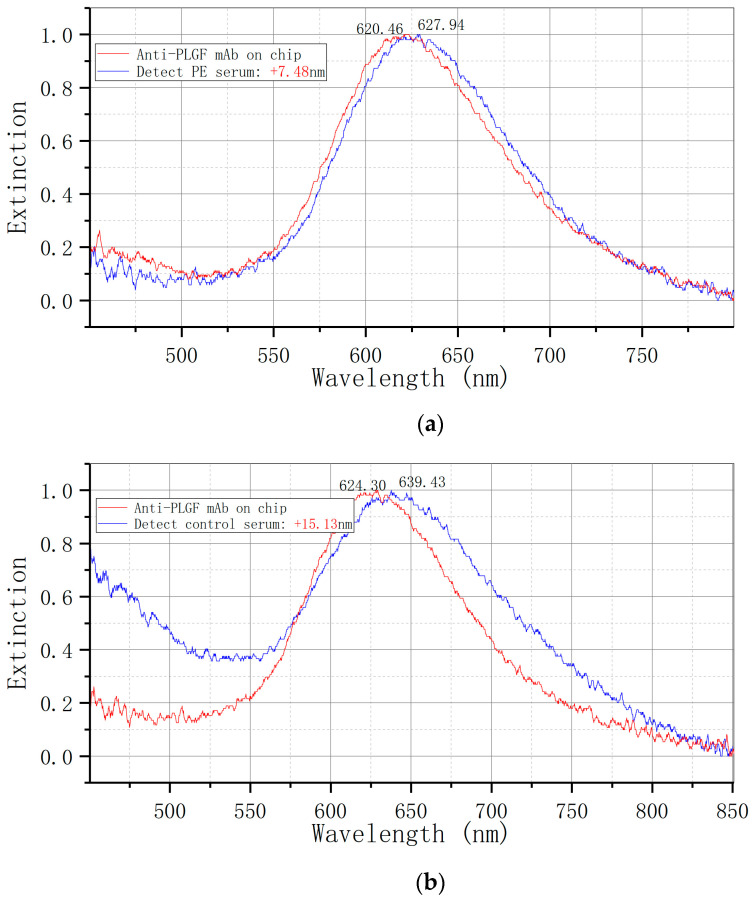
Detection of serum PLGF expression by LSPR bowtie sensor: (**a**) PE group (Δλ = +7.48 nm); (**b**) control group (Δλ = +15.13 nm). mAb: monoclonal antibody.

The bowtie LSPR biosensor was employed to assess serum placental growth factor (PLGF) expression in two cohorts of pregnant women. Figure 7 illustrates that the elimination peak of the preeclampsia (PE) group shifted from 620.46 nm to 627.94 nm, resulting in a Δλ of +7.48 nm, while the control group exhibited a redshift from 624.30 nm to 639.43 nm, corresponding to a Δλ of +15.13 nm.

An independent sample t-test was utilized to analyze the data, with the results presented in Table 1. The LSPR biosensor was employed to detect the extinction signal of serum samples (Δλmax). The extinction signal of serum sFlt-1 in the preeclampsia group was found to be significantly higher than that in the normal pregnancy group (14.37 ± 2.56 nm vs. 4.21 ± 1.36 nm), with a *p*-value of 0.008. Similarly, the serum PLGF extinction signal in the preeclampsia group was significantly lower than that in the normal pregnancy group (5.36 ± 3.15 nm vs. 11.47 ± 4.92 nm), with a *p*-value of 0.013.

**Figure 8:**
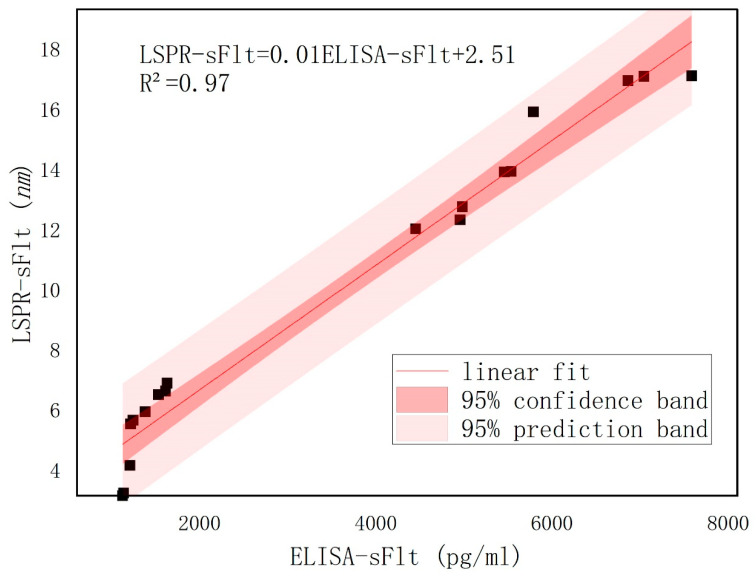
Parallel detection of serum sFlt-1 by ELISA kit and bowtie nanoscale LSPR biosensor.

**Figure 9:**
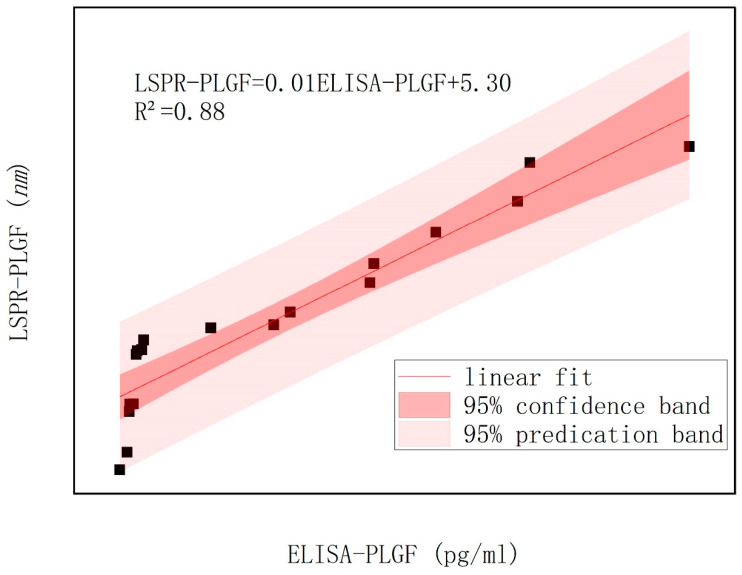
Serum PLGF was detected by ELISA kit and bowtie nanoscale LSPR biosensor.

To assess the reliability of the BNA LSPR biosensor developed by the research team, both an ELISA kit and the LSPR bowtie biosensor were utilized to concurrently measure sFlt-1 (Figure 8) as well as PLGF (Figure 9) expression in serum samples from patients. The linear equation of s-Flt was LSPR-sFlt (nm) = 0.01 × [ELISA-sFlt] (pg/mL) + 2.51, with an R² values of 0.97, while the linear equation of PLGF was LSPR-PLGF (nm) = 0.01 × [ELISA-PLGF] (pg/mL) + 5.30, with an R² values of 0.88. The correlation analysis findings between the LSPR and ELISA methods were congruent, indicating a linear association. These results suggest promising clinical detection capabilities for the bowtie nanoscale LSPR biosensor. Figure 8 and Figure 9 demonstrated that deviations fall within a 95% confidence interval, ensuring reliable interpretation of results.

## 4. Discussion

Currently, there is a trend of delaying childbearing age, which has been associated with a higher incidence of preeclampsia and adverse perinatal outcomes. Early prediction of preeclampsia and timely intervention are crucial strategies for mitigating negative outcomes for both mothers and infants [4,5]. The bowtie nano-optical antenna is capable of generating local surface plasmon resonance when exposed to specific wavelengths of light, resulting in enhanced dipole effects and increased electromagnetic fields, thereby enabling its unique biosensing capabilities [23,24]. To our best knowledge, there are no reports of clinical disease detection using BNA LSPR sensors. This study utilized the coupling effect of the electric field at the gap of a bowtie structure, the substantial enhancement of local electric field strength and the enhancement of refractive index sensitivity near the interface to investigate chemical modification techniques in conjunction with simulation outcomes. Various probes were tailored on the bowtie surface to enable the simultaneous detection of multiple specific molecular markers. This work establishes an experimental basis and provides data support for the potential application of BNA LSPR sensors in the early detection of clinical diseases at trace levels.

The sensitivity of the sensing structure was analyzed through the utilization of FDTD and DDA algorithms, which involved modifying the material composition, shape and structure arrangement of metal nanostructures based on the local coupled electric field strength. This approach was employed to design a BNA structure with optimal LSPR response. As shown in Figure 1, the antenna structures of circular dimer, quadrilateral dimer and triangular dimer (bowtie) metal nanoparticles are simulated and designed. The findings presented in Figure 2 demonstrate that the BNA structure exhibits a significant field enhancement, attributed to the synergistic interaction between the local surface plasmon resonance of the nanoantennas and their image modes within the gold film. This approach to field enhancement circumvents the conventional difficulties associated with fabricating minuscule nanotips or nanogaps, offering a straightforward method for developing sophisticated substrates with expansive surface areas, heightened sensitivity, and enhanced reliability.

The study utilized a mercaptoundecanoic acid (MUA) amino conjugated membrane [17,19] to biofunctionalize bowtie gold nanoparticles, immobilize sFlt-1 and PLGF monoclonal antibodies, and fabricate biosensor chips. By employing an LSPR biosensor chip to analyze serum samples from preeclampsia patients and normal pregnant women, a notable disparity in extinction signal was observed. This discrepancy in extinction signal between the two cohorts was found to be statistically significant, underscoring the efficacy of the bowtie nanosensor chip in distinguishing preeclampsia.

While the commercially available ELISA kit provides a viable semi-quantitative measurement, it has several limitations, including low detection efficiency, prolonged detection time and the requirement for potentially harmful labeling. Our proposed LSPR method, although currently limited by sample size and unable to achieve full quantitative analysis, offers distinct advantages: remarkable sensitivity, rapid detection without the need for toxic molecular labeling and reliance on optical signal changes. Looking ahead, increasing the sample size will enable the development of an LSPR standard curve for quantitative detection. Furthermore, enhancing nanostructure and biosensitive membrane technologies can further boost sensitivity. The integration of microfluidic technology promises simultaneous detection of multiple markers, enhancing accuracy and paving the way for high-throughput, efficient detection.

## 5. Conclusions

This study establishes the experimental groundwork and data validation for utilizing BNA LSPR sensors in the early detection of clinical diseases, particularly offering a novel approach for predicting preeclampsia. The LSPR biosensor, employing bowtie nanoparticles, demonstrates a high sensitivity, label-free detection and rapid detection in identifying serum placental circulating factors associated with preeclampsia, thus presenting a promising method for early preeclampsia detection. Future research opportunities include increasing the sample size to develop a standard curve for quantitative detection, integrating microfluidic technology, which promises simultaneous detection of multiple markers for high-throughput detection, and conducting prospective cohort studies to further assess the real-world effectiveness of BNA LSPR biosensors in predicting PE.

## Figures and Tables

**Table 1:** Detection of placental angiogenesis-related factors in serum of patients with preeclampsia by LSPR sensor.

Δλmax (nm)	PE Group (n_1_ = 9)	Normal Group (n_2_ = 9)	t	Sig. (Two-Tail)	95% CI
sFlt-1	14.37 ± 2.56	4.21 ± 1.36	2.742	0.008	7.35~19.54
PLGF	5.36 ± 3.15	11.47 ± 4.92	2.694	0.013	6.71~20.05

Note: Δλmax: drift of the extinction pick; Sig: *p* < 0.05, indicating a statistically significant difference.

## Data Availability

The original contributions presented in the study are included in the article, further inquiries can be directed to the corresponding author.

## Abbreviations

LSPR	localized surface plasmon resonance
FDTD	finite-different time-domain
DDA	discrete dipole approximation
EBL	electron beam lithography
BNA	bowtie nanoantenna
sFlt-1	soluble vascular endothelial growth factor receptor 1
PLGF	placental growth factor
ELISA	enzyme-linked immune-adsorbent assay
PE	preeclampsia
MUA	11-mercaptoundecanoic acid
NHS	N-hydroxysuccinimide
EDC	1-(3-dimethylaminopropyl) -3-ethylcarbodiimide
BMI	body mass index
EDTA	ethylene diamine tetraacetic acid
EBL	electron beam lithography
PMMA	polymethyl methacrylate
CCD	charge coupled device
OD	optical density
SEM	scanning electron microscope
